# Effect of Surgeon-Performed Thoracic Paravertebral Block on Postoperative Pain in Adolescent Idiopathic Scoliosis Surgery: A Prospective Randomized Controlled Trial

**DOI:** 10.3390/jpm14060659

**Published:** 2024-06-20

**Authors:** Bora Lee, Eun Jung Kim, Jin Ha Park, Kun-Bo Park, Yong Seon Choi

**Affiliations:** 1 Department of Anesthesiology and Pain Medicine, Severance Hospital and Anesthesia and Pain Research Institute, Yonsei University College of Medicine, 50-1 Yonsei-ro, Seodaemun-Gu, Seoul 03722, Republic of Korea; 2 Department of Orthopedic Surgery, Severance Hospital, Yonsei University College of Medicine, Seoul 03722, Republic of Korea

**Keywords:** nerve block, pain management, pediatrics, scoliosis, spine

## Abstract

Posterior spinal fusion for adolescent idiopathic scoliosis (AIS) causes severe postoperative pain. Thoracic paravertebral block (PVB) provides excellent analgesia during various surgeries. We examined the effects of PVB on postoperative analgesia in children undergoing AIS surgery. In this study, 32 children scheduled for AIS surgery were randomly assigned to receive either PVB (PVB group) or no block (control group). The PVB group underwent surgeon-performed PVB with 0.5 mL/kg of adrenalized 0.2% ropivacaine on each side. The primary outcome was the pain score at rest at 6 h postoperatively. Secondary outcomes included pain scores both at rest and during movement and analgesic use for 48 h postoperatively. The postoperative resting pain scores at 6 h were comparable between the control and PVB groups (5.2 ± 2.0 and 5.1 ± 1.8, respectively), with no significant differences. However, at 1 h postoperatively, the control group showed significantly higher resting and mean moving pain scores than the PVB group (*p* < 0.05). The pain scores at other time points and analgesic use were comparable between the groups. Initial benefits of surgeon-performed bilateral PVB were observed but diminished at 6 h postoperatively. Future research using various anesthetics is needed to extend the effects of PVB.

## 1. Introduction

Adolescent idiopathic scoliosis (AIS) is the most prevalent type of scoliosis and typically manifests at approximately 11–18 years of age, with an occurrence rate of 0.47–5.2% [1]. It is characterized by multifactorial influences involving changes in intrinsic (genetics, hormones, tissues, neurology, and spine biomechanics) and extrinsic (lifestyle and environment) factors [2]. The severity of AIS is gauged by the Cobb angle, which is measured at the curvature between the upper and lower vertebrae in the frontal plane [3]. Cobb angles exceeding approximately 30° and 50° at skeletal maturity heighten the risk of cardiopulmonary distress, cosmetic deformity, and persistent pain, leading to functional limitations in adulthood [4,5]. Therefore, the correction of AIS with a severe Cobb angle is crucial for enhancing quality of life [6]. The most common complication of this surgery is surgical infection (approximately 2%), and this surgical procedure showed favorable long-term functional results, even generally allowing a full return to sport [7,8].

The decision to perform surgical intervention for a patient with AIS depends on various factors, including the size and pattern of the curve, the progression of the curve, and skeletal maturity. For skeletally immature patients with structural thoracic curves measuring over 40° or those who continue to progress, surgery is typically considered [1]. According to survival analysis, approximately 0.7% of diagnosed patients undergo surgical treatment within five years [9]. The main surgical steps of posterior spinal fusion surgery for AIS (AIS surgery) include subperiosteal muscle dissection, facetectomy/decortication, pedicle screw insertion, rod placement, derotation, and correction maneuvers [7]. This surgical procedure involves bilaterally inserting metal rods adjacent to the spine, which are attached using hooks or screws and then rotated to realign and maintain the new alignment of the spine [10]. Therefore, patients often experience severe pain after undergoing AIS surgery [11]. AIS surgery involves extensive surgical incisions, stimulating nociceptors within densely innervated periarticular tissues, thereby inducing continuous deep somatic pain and severe reflex spasms of the paraspinal muscles [3]. However, research on pain management during AIS surgery is lacking.

An effective pain management strategy for AIS surgery can be achieved through the use of a multimodal approach that incorporates patient-controlled analgesia (PCA), nonsteroidal anti-inflammatory drugs, paracetamol, and local anesthetic infiltration [10,12]. Inadequate pain management after AIS surgery in pediatric patients often requires the use of opioid analgesics [13]. A recent study on the management of postoperative pain in pediatric patients who underwent spinal surgery discovered that mitigating adverse effects associated with opioid use, including nausea and constipation, can facilitate prompt resumption of functional activity [10]. In addition, opioid use for pain control can delay postoperative recovery and increase the risk of opioid use disorders, particularly in children [14]. Therefore, regional analgesia emerges as a pivotal strategy for reducing postoperative pain and minimizing the need for opioids, promoting recovery. In particular, during AIS surgery, exposure of the structures around the spine allows surgeons to administer paravertebral blocks (PVBs) easily, presenting an effective alternative for pain management. Numerous studies have reported the analgesic effects of PVBs after various types of surgery; however, research on the use of PVBs in spinal surgery remains limited [15,16,17,18]. Therefore, this study aimed to evaluate the effectiveness of intraoperative surgeon-performed PVBs in relieving pain in pediatric patients following AIS surgery.

## 2. Materials and Methods

This prospective randomized controlled study received approval from the Severance Hospital Institutional Review Board (protocol number: 4-2020-1467) on 22 February 2021 and was registered on ClinicalTrials.gov (NCT04773509, 26 February 2021). This study adhered to the Consolidated Standards for Reporting Trials and Guidelines for Clinical Trials. We enrolled 32 children aged 10–18 years who were scheduled to undergo elective AIS surgery between 24 June 2021 and 24 December 2023. The exclusion criteria were as follows: allergy to local anesthetics and inability to understand the study or express their level of pain. Parents granted written informed consent, and assent was obtained from the child or adolescent patient when appropriate.

The patients were randomly allocated to either receive PVB (PVB group) or no block (control group) based on a random computer-generated sequence on the day of surgery. Allocation was conducted by an investigator not involved in anesthesia administration, perioperative care, or postoperative outcome evaluation. The anesthesiologists who provided anesthesia did not participate in the data collection. Healthcare providers, patients, and researchers responsible for follow-up and data collection were blinded to the group allocation assignments.

### 2.1. Standard of Care

Routine monitoring was performed upon patient arrival in the operating room. General anesthesia was induced using propofol with an effect-site concentration of 4.0–5.0 mcg/mL, combined with remifentanil at an effect-site concentration of 3.0–3.5 ng/mL. This was followed by the administration of rocuronium 0.6 mg/kg to facilitate tracheal intubation. Anesthesia was maintained using propofol and remifentanil, with a target patient state index of 25–50. Subsequently, under ultrasound guidance, a central venous catheter was inserted into the internal jugular vein, and an arterial cannula was inserted into the left radial artery. Hypotension was defined as a systolic arterial pressure <90 mmHg and was treated with fluid therapy and/or continuous infusion of norepinephrine.

All anesthetized patients were transferred to a Jackson table in the prone position. Intraoperative somatosensory- and transcranial motor-evoked potentials were monitored. Following the standard posterior approach, a guide pin for the pedicle screw was inserted using the posteroanterior image-intensifier rotation technique. Around the apex, posterior column osteotomy was performed for the rigid curves. The rod was contoured according to the scoliotic curvature. Pedicle screws were inserted at each level, and the rods were connected. Derotation of the long rod was performed to treat thoracic kyphosis and lumbar lordosis. Each curve was distracted or compressed. Direct vertebral rotation was performed at the apex, uppermost instrumented vertebra, or lowermost instrumented vertebra, according to the severity of the rotation. Sequential decortication of the lamina and bone grafting were performed, and the wound was closed. A single surgical team performed all surgical procedures. All patients received an intravenous infusion of acetaminophen at a dose of weight × 15 mg and dexamethasone at a dose of weight × 0.1 mg during the surgical procedure. Tranexamic acid was administered at a dose of 10 mg per body weight for 1 h, followed by intravenous infusion at a rate of 1 mg/kg/h until the end of the procedure.

For the management of postoperative pain, an intravenous PCA pump was initiated at the end of surgery in all patients. The PCA pump contained fentanyl (Hana Pharm. Co., Ltd., Seoul, Republic of Korea) 18 μg/kg (total volume including saline: 120 mL), infused at 1 mL/h with a 1 mL bolus dose and 15 min lockout time. Every 12 h, each patient received an intravenous dose of acetaminophen (15 mg/kg) as a component of the protocol for managing postoperative pain. Intravenous tramadol (1 mg/kg) was administered as a rescue analgesic when the numeric pain score (NRS) was >4.

### 2.2. Surgeon-Performed Paravertebral Block

**Figure 1:**
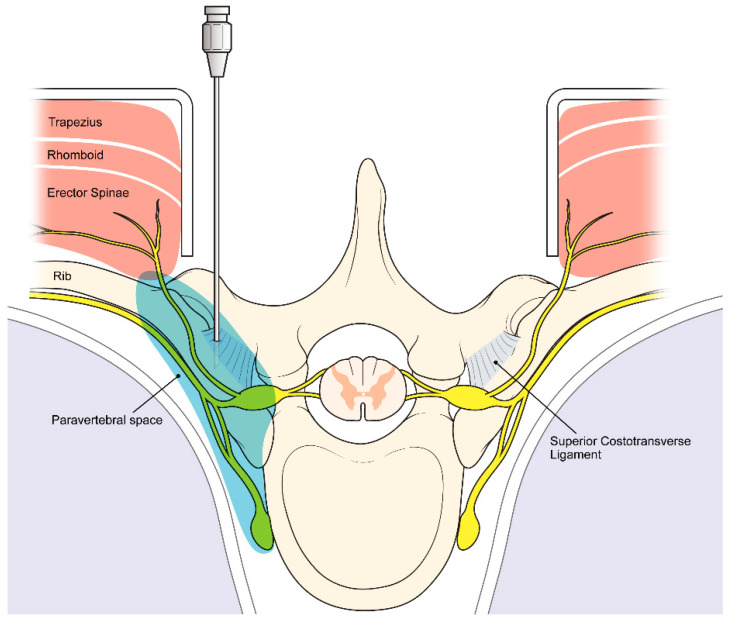
Paravertebral block (PVB) is performed by an orthopedic surgeon during surgery. A 22-gauge needle is advanced to the lower aspect of the transverse process before pedicle screw insertion. After perforating the superior costotransverse ligament and following negative aspiration, anesthesia is injected into the paravertebral space.

In the PVB group, a surgeon performed PVB under direct vision before pedicle screw insertion. A 22-gauge needle was advanced to the lower aspect of the transverse process (Figure 1). After perforating the superior costotransverse ligament and following negative aspiration, a total of 0.5 mL/kg of 0.2% ropivacaine with 1:200,000 epinephrine was injected into the paravertebral space at the T3/4, T6/7, and T9/10 thoracic interspaces. The PVB was performed on the opposite side using the same methodology. All the patients received ropivacaine at a dose not exceeding 3 mg/kg.

### 2.3. Outcome Assessments

The primary outcome was the pain score at rest 6 h after surgery. The secondary endpoints were pain scores both at rest and during movement at other time points, fentanyl consumption, and the need for rescue analgesics. The severity of pain at rest and during movement was determined using an 11-point NRS (0 = no pain; 10 = worst imaginable pain) [19]. Pain scores were recorded at six time points: preoperative baseline and at 1, 6, 12, 24, and 48 h postoperatively (Supplemental Figure S1). Data collected from the PCA pump included fentanyl consumption, the number of boluses administered, and invalid bolus attempts.

### 2.4. Statistical Analysis

The calculation of the necessary sample size to detect a difference in pain scores of more than one unit between the control and PVB groups was performed [20]. Based on this calculation, it was determined that 14 participants were required in each group to achieve a statistical power of 90% at a significance level of *p* < 0.05. To accommodate a dropout rate of 10%, a total of 16 patients were enrolled in each group. The assumptions of parametricity were confirmed using Shapiro–Wilk and Kolmogorov–Smirnov tests. Parametric continuous variables were analyzed using the independent *t*-test, whereas non-parametric continuous variables were analyzed using the Mann–Whitney *U* test. Intergroup comparisons were conducted using the *t*-, Mann–Whitney *U*, Fisher’s exact, or χ^2^ test, as appropriate. Continuous variables are presented as mean ± SD or median (interquartile range), whereas categorical variables are presented as numbers (percentages). Linear mixed models were applied to repeatedly measure pain scores with group, time, and group-by-time fixed effects. Post hoc analysis was conducted using the Bonferroni correction to account for multiple comparisons. To calculate effect sizes, Cohen’s d was used for independent *t*-tests, Wilcoxon effect size (r) was used for Mann–Whitney *U* tests, and Cramer’s V was used for Fisher’s exact tests or chi-square tests (Supplemental Tables S1 and S2). For an independent *t*-test, the *t* statistic and degrees of freedom (df) are reported. For a chi-square test, the χ^2^ statistic and df are documented. For a Mann–Whitney *U* test, the *U*-value is reported, with df denoted as not applicable (NA). For a Fisher exact test, both the statistic and df are denoted as NA. Statistical analyses were performed using R version 4.1.1 (R Foundation for Statistical Computing, Vienna, Austria), IBM SPSS Statistics for Windows (version 23.0; IBM Corp., Armonk, NY, USA), and MedCalc Statistical Software version 18.11.3 (MedCalc Software Ltd., Ostend, Belgium). The level of statistical significance was set at *p* < 0.05.

## 3. Results

**Figure 2:**
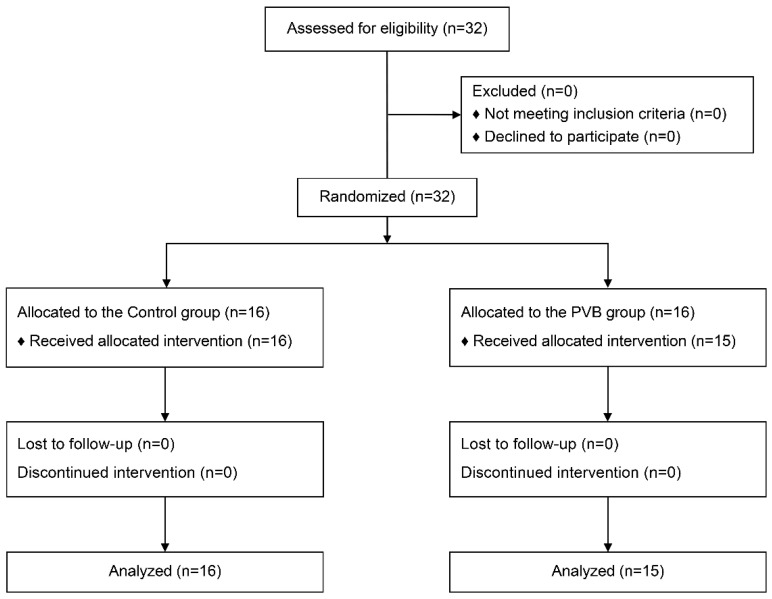
CONSORT study flow diagram. CONSORT, Consolidated Standards for Reporting Trials. PVB, paravertebral block.

Among the 32 individuals who underwent screening for eligibility, all were enrolled and assigned to either the PVB or control group. However, one patient in the PVB group was excluded because of the challenging nature of PVB, which ultimately failed. Accordingly, data from 31 patients were examined in the final analysis. Figure 2 presents a flowchart of the study participants.

No significant differences were observed in patient characteristics between the two groups (Table 1). Operative variables, including the Cobb angle, bending, flexibility, and vertebral levels at which surgery was performed, and correction rates were comparable between the two groups. The amount of bleeding was greater in the PVB group than in the control group (687 vs. 456 mL, *p* = 0.037). The amount of transfusion was also higher in the PVB group; however, the difference was not statistically significant. The total amount of norepinephrine administered was also higher in the PVB group than in the control group; however, this difference was not statistically significant.

**Figure 3:**
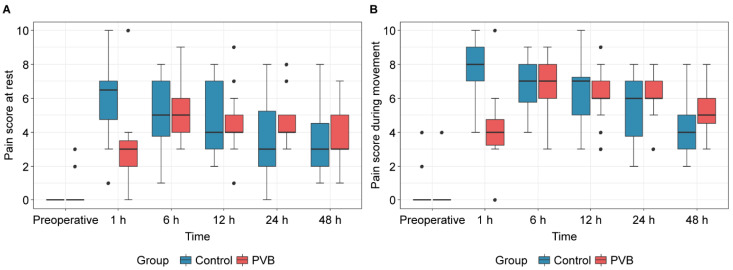
(**A**) Pain scores at rest and (**B**) during movement. Boxplots display the median with the 25th/75th percentiles. Whiskers indicate the minimum/maximum values, excluding outliers. Points on the plot represent the outliers. PVB, paravertebral block.

**Figure 4:**
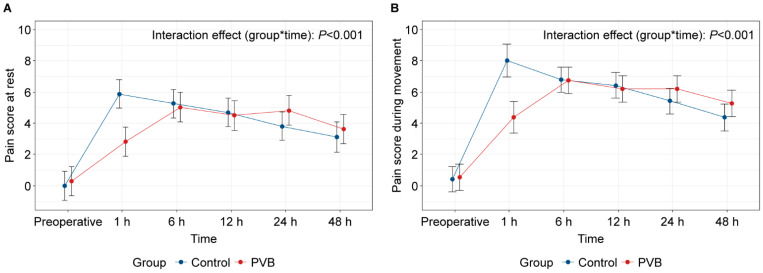
(**A**) Changes in pain scores for 48 h postoperatively at rest and (**B**) during movement. Values are the estimated means and 95% confidence intervals, analyzed using a linear mixed-effects model for each time point. PVB, paravertebral block.

The mean scores for postoperative resting pain at 6 h were 5.2 ± 2.0 and 5.1 ± 1.8 in the control and PVB groups, respectively, demonstrating no statistical difference (*p* = 0.795, Figure 3). Similarly, there were no significant differences in the resting and moving pain scores at 12, 24, and 48 h postoperatively. However, at 1 h post-surgery, both resting and moving pain scores were significantly higher in the control group than in the PVB group (median resting pain, 6.5 vs. 3.0, *p* = 0.001; mean moving pain, 7.8 vs. 4.3, *p* = 0.005). Therefore, a significant difference was noted over time in postoperative changes in pain scores between the two groups (Figure 4; *P*_group*time_ < 0.001).

Fentanyl consumption as a background infusion plus boluses via a PCA pump and the number of patients receiving rescue analgesics at any time point were comparable between the two groups (Table 2). However, the proportion of invalid boluses to the patient’s total number of attempts on the PCA machine tended to be higher in the control group than in the PVB group (44% vs. 27%, *p* = 0.093). The number of boluses administered with PCA during the postoperative 1 h was also higher in the control group than in the PVB group (2 vs. 1, *p* = 0.05). There was no statistical difference in fentanyl consumption at 1–6 h postoperatively between the two groups; however, it was higher in the PVB group.

## 4. Discussion

To the best of our knowledge, this trial is the first randomized trial to compare pain scores between control and PVB groups in patients undergoing AIS surgery. The pain score at 1 h postoperatively was significantly lower in the PVB group; however, this difference was no longer significant at 6 h postoperatively.

Severe pain after spine surgery is relatively common, and if pain is inadequately controlled, it can impede postoperative recovery [21]. Moreover, AIS surgery involves a wide incision and the manipulation of multiple spinal levels, causing intense postoperative pain [22]. However, only a few studies have been conducted on postoperative pain control, particularly in pediatric patients undergoing AIS surgery [13,20,23].

The thoracic paravertebral space is enclosed by the parietal pleura on the anterolateral side and by the transverse processes along the vertebral bodies (T1 to L1) on the posteromedial side [24]. The paravertebral space contains the anterior and posterior branches of the spinal nerves, intercostal spinal nerves, and the sympathetic chain. In addition, the thoracic paravertebral space is connected laterally to the intercostal space, the epidural space through the intervertebral foramina, and the contralateral paravertebral space across the prevertebral and epidural spaces [25]. In a previous study, local anesthetics had extended outside the paravertebral space after PVB in 40% of cases [26]. Therefore, PVB is effective for somatic analgesia during thoracic, cardiac, chest wall, and breast surgeries [27,28,29,30]. In particular, the superior costotransverse ligament, extending from the lower edge of the transverse process to the upper edge of the rib beneath, constitutes the posterior boundary of the paravertebral space [15]. Due to this anatomical configuration, during AIS surgery, the structures around the thoracic vertebrae are exposed, providing surgeons with enhanced visualization and easier access to the paravertebral space. Recently, PVB using ultrasonography has been widely performed [31]; however, this procedure can be challenging in pediatric patients with scoliosis. In contrast, this visibility is particularly important when PVB is performed during AIS surgery, as it enables the surgeon to accurately and effectively address spinal deformities.

In pediatric patients, PVB has demonstrated efficacy in relieving postoperative pain following various surgical procedures, including open cardiac surgery, thoracotomy, and abdominal operations, similar to that in adults [32,33,34,35]. The analgesic effect of PVB has also been studied in spinal surgery. Alansary et al. revealed that bilateral trans-incisional PVB with dexamethasone led to a reduction in opioid consumption [36]. The erector spinae plane block, which works in a mechanism similar to that of PVB, has been well studied in spinal surgery, with common findings of lower postoperative pain scores and reduced opioid use [37,38,39]. Based on previous studies, we expect the analgesic effects of PVB to be effective in pediatric patients undergoing AIS surgery. Recently, there have been several studies on the effectiveness of PVBs performed by surgeons during minimally invasive thoracic surgery [40,41]. The effectiveness of PVBs performed by surgeons during thoracoscopic procedures is comparable to that of PVBs performed by anesthesiologists under ultrasound guidance [40]. Similarly, no statistical differences in pain or opioid use were observed between patients who received a video-assisted paravertebral catheter placed by the surgeon and those who received an anesthesiologist-administered ESP catheter [41]. Therefore, we aimed to evaluate the analgesic efficacy of intraoperative surgeon-performed PVB after AIS surgery, advocating for further research on the effectiveness of surgeon-performed PVB in spinal surgery.

Our results demonstrated that the PVB group had a significantly lower pain score than the control group 1 h after surgery. However, 6 h after surgery, the pain scores between the two groups were not significantly different. Although there was no statistical difference in fentanyl consumption 1–6 h postoperatively between the two groups, it is likely that this rebound pain was the reason why fentanyl consumption was higher in the PVB group. This also could be attributed to the limited coverage of the extensive surgical area by the PVB, as its spread of local anesthetics in the craniocaudal direction is unpredictable [25,26]. The PVB was performed approximately 1 h after the start of surgery; considering the limited duration of action of ropivacaine and the longer duration of AIS surgery, it is unlikely that analgesia would have lasted until 6 h postoperatively, which is approximately 10 h after the PVB. Furthermore, we used 0.2% ropivacaine for the PVB to prevent motor block [42]. Although no significant difference was noted in pain intensity, the control group had more invalid bolus attempts on PCA within 48 h after AIS surgery than the PVB group. These results imply that patients in the control group experienced more frequent and intense pain perception than those in the PVB group. Further studies on PVB with higher concentrations or volumes of anesthetics are warranted.

This study yielded some substantial insights into the effectiveness of PVB for AIS surgery; however, it has some limitations. First, the number of enrolled patients was small, reflecting the scarcity of prior research on pain management in AIS surgery, thereby underscoring the need for additional investigation. Second, the control group lacked a sham block for direct comparison with the PVB group. Third, PVB was performed using adrenalized 0.2% ropivacaine (1 mL/kg) in this study. The clinical effect of PVB may vary depending on the concentration and volume of the local anesthetic. Finally, while the number of vertebral levels at which surgery was performed did not differ significantly between the two groups, more spinal levels were operated on in the PVB group. As a result, intraoperative blood loss and blood transfusions were higher in the PVB group than in the control group. Although the group allocation was randomized to reduce this difference, the small number of patients in the study may have contributed to this difference.

## 5. Conclusions

This study showcased that intraoperative surgeon-performed bilateral thoracic PVB provided effective postoperative analgesia in pediatric patients undergoing AIS surgery. However, this analgesic effect was not sustained beyond 6 h postoperatively. Further research is necessary to explore the effect of increased concentrations and volumes of anesthetics used for PVB.

## Figures and Tables

**Table 1:** Demographic and operative variables.

	Control Group (N = 16)	PVB Group (N = 15)	*p* Value
Demographic data			
Age (years)	14.1 ± 2.6	13.7 ± 1.9	0.694
Female/Male	11/5	11/4	>0.999
Height (cm)	156.6 ± 11.4	160.9 ± 13.0	0.332
Weight (kg)	48.4 ± 8.7	52.5 ± 14.8	0.364
Body mass index (kg/m^2^)	19.7 ± 2.6	20.1 ± 4.1	0.764
ASA class (I/II/III)	8/6/2	10/3/2	0.686
Preoperative Cobb angle	56.5 [51.5–71.0]	59.0 [55.5–61.5]	0.566
Preoperative bending	30.9 ± 15.5	30.9 ± 16.0	0.999
Flexibility	48.8 ± 26.5	50.2 ± 23.1	0.870
Duration of surgery (min)	249.0 [196.0–354.0]	273.0 [246.5–300.5]	0.406
Surgical vertebral level during surgery	8.0 [7.0–13.0]	11.0 [9.0–11.0]	0.425
Postoperative Cobb angle	20.2 ± 11.0	20.0 ± 9.7	0.947
Correction rate	66.6 ± 18.6	67.6 ± 13.7	0.862
Anesthesia duration (min)	340.0 [300.0–457.5]	375.0 [340.0–417.5]	0.313
Remifentanil use (mcg)	2694.6 ± 826.9	2856.4 ± 1231.2	0.687
Propofol use (mg)	2113.0 [1908.5–2315.0]	2110.0 [1910.0–3305.5]	0.478
Norepinephrine use (mcg)	18.5 [4.4–36.0]	36.2 [24.7–63.5]	0.163
Amount of fluid infused (mL)	2342.5 ± 794.2	2684.7 ± 832.0	0.251
Amount of bleeding (mL)	456.4 ± 245.4	686.7 ± 336.2	0.037
Amount of transfusion (mL)	252.4 ± 254.9	394.0 ± 160.4	0.077

Values are reported as the median (interquartile range), mean ± standard deviation, or number of patients (%). ASA, American Society of Anesthesiologists.

**Table 2:** Intravenous patient-controlled and postoperative rescue analgesia.

	Control Group (N = 16)	PVB Group (N = 15)	*p* Value
Intravenous PCA data			
Fentanyl consumption (μg) as background infusion plus boluses via PCA			
0–1 h	20.3 ± 9.9	16.5 ± 10.2	0.313
1–6 h	78.2 [57.2–92.5]	96.7 [64.8–130.3]	0.338
6–12 h	72.5 [49.9–107.5]	67.7 [51.0–121.3]	0.859
12–24 h	133.8 [95.5–212.4]	125.7 [94.1–211.8]	0.953
24–48 h	282.2 ± 147.7	291.8 ± 81.1	0.823
Number of boluses given via PCA			
0–1 h	2 (1–3)	1 (0–2)	0.050
1–6 h	6.6 ± 3.4	8.3 ± 4.4	0.237
6–12 h	5.0 [2.5–8.0]	4.0 [1.5–9.5]	0.983
12–24 h	8.0 [2.5–20.0]	5.0 [3.0–13.0]	0.708
24–48 h	13.1 ± 11.9	13.7 ± 8.3	0.886
Number of invalid bolus attempts via PCA			
0–1 h	3.5 [0.0–6.0]	1.0 [0.0–3.0]	0.267
1–6 h	3.0 [0.5–11.5]	1.0 [0.0–10.5]	0.556
6–12 h	0.0 [0.0–4.0]	0.0 [0.0–6.0]	0.982
12–24 h	1.0 [0.0–10.5]	0.0 [0.0–4.0]	0.485
24–48 h	1.0 [0.0–3.0]	0.0 [0.0–3.0]	0.804
Patients receiving rescue analgesics (n)			
0–1 h	13 (81%)	10 (67%)	0.433
1–6 h	7 (44%)	7 (47%)	>0.999
6–12 h	2 (13%)	5 (33)	0.220
12–24 h	3 (19%)	6 (40%)	0.252
24–48 h	6 (38%)	10 (67%)	0.206

Values are presented as median (interquartile range), mean ± standard deviation, or number of patients (%). PCA, intravenous patient-controlled analgesia.

## Data Availability

The data that support the outcomes of this study can be provided by the corresponding author upon request.

## Supplementary Materials

The following supporting information can be downloaded at https://www.mdpi.com/article/10.3390/jpm14060659/s1: Figure S1: Study timeline. Table S1: Demographic and operative variables. Table S2: Intravenous patient-controlled and postoperative rescue analgesia.

## References

[1] Addai D., Zarkos J., Bowey A.J. (2020). Current concepts in the diagnosis and management of adolescent idiopathic scoliosis. Childs Nerv. Syst..

[2] Peng Y., Wang S.R., Qiu G.X., Zhang J.G., Zhuang Q.Y. (2020). Research progress on the etiology and pathogenesis of adolescent idiopathic scoliosis. Chin. Med. J..

[3] Borgeat A., Blumenthal S. (2008). Postoperative pain management following scoliosis surgery. Curr. Opin. Anaesthesiol..

[4] Negrini S., Donzelli S., Aulisa A.G., Czaprowski D., Schreiber S., de Mauroy J.C., Diers H., Grivas T.B., Knott P., Kotwicki T. (2018). 2016 SOSORT guidelines: Orthopaedic and rehabilitation treatment of idiopathic scoliosis during growth. Scoliosis Spinal Disord..

[5] Tambe A.D., Panikkar S.J., Millner P.A., Tsirikos A.I. (2018). Current concepts in the surgical management of adolescent idiopathic scoliosis. Bone Jt. J..

[6] Mens R.H., Bisseling P., de Kleuver M., van Hooff M.L. (2022). Relevant impact of surgery on quality of life for adolescent idiopathic scoliosis: A registry-based two-year follow-up cohort study. Bone Jt. J..

[7] Yang H., Jia X., Hai Y. (2022). Posterior minimally invasive scoliosis surgery versus the standard posterior approach for the management of adolescent idiopathic scoliosis: An updated meta-analysis. J. Orthop. Surg. Res..

[8] Barone G., Giudici F., Manzini F., Pironti P., Viganò M., Minoia L., Archetti M., Zagra A., Scaramuzzo L. (2023). Adolescent idiopathic scoliosis surgery: Postoperative functional outcomes at 32 years mean follow-up. Children.

[9] Sung S., Chae H.W., Lee H.S., Kim S., Kwon J.W., Lee S.B., Moon S.H., Lee H.M., Lee B.H. (2021). Incidence and surgery rate of idiopathic scoliosis: A nationwide database study. Int. J. Environ. Res. Public Health.

[10] Young C.D., McLuckie D., Spencer A.O. (2019). Anaesthetic care for surgical management of adolescent idiopathic scoliosis. BJA Educ..

[11] Bailey K.M., Howard J.J., El-Hawary R., Chorney J. (2021). Pain trajectories following adolescent idiopathic scoliosis correction: Analysis of predictors and functional outcomes. JBJS Open Access.

[12] Locke L.L., Rhodes L.N., Sheffer B.W. (2023). Accelerated protocols in adolescent idiopathic scoliosis surgery. Orthop. Clin..

[13] Lee C.S., Merchant S., Chidambaran V. (2020). Postoperative pain management in pediatric spinal fusion surgery for idiopathic scoliosis. Paediatr. Drugs.

[14] Nelson R., Shimon T., Grimsby G.M. (2021). Pediatric urologic surgery: Reducing opioid use. Paediatr. Drugs.

[15] Pawa A., Wojcikiewicz T., Barron A., El-Boghdadly K. (2019). Paravertebral blocks: Anatomical, practical, and future concepts. Curr. Anesthesiol. Rep..

[16] Yeung J.H., Gates S., Naidu B.V., Wilson M.J., Gao Smith F. (2016). Paravertebral block versus thoracic epidural for patients undergoing thoracotomy. Cochrane Database Syst. Rev..

[17] Terkawi A.S., Tsang S., Sessler D.I., Terkawi R.S., Nunemaker M.S., Durieux M.E., Shilling A. (2015). Improving analgesic efficacy and safety of thoracic paravertebral block for breast surgery: A mixed-effects meta-analysis. Pain Physician.

[18] El-Boghdadly K., Madjdpour C., Chin K.J. (2016). Thoracic paravertebral blocks in abdominal surgery—A systematic review of randomized controlled trials. Br. J. Anaesth..

[19] Almarzouki A.F., Brown C.A., Brown R.J., Leung M.H.K., Jones A.K.P. (2017). Negative expectations interfere with the analgesic effect of safety cues on pain perception by priming the cortical representation of pain in the midcingulate cortex. PLoS ONE.

[20] Erdogan M.A., Ozgul U., Ucar M., Korkmaz M.F., Aydogan M.S., Ozkan A.S., Colak C., Durmus M. (2017). Patient-controlled intermittent epidural bolus versus epidural infusion for posterior spinal fusion after adolescent idiopathic scoliosis: Prospective, randomized, double-blinded study. Spine.

[21] Puvanesarajah V., Liauw J.A., Lo S.F., Lina I.A., Witham T.F., Gottschalk A. (2015). Analgesic therapy for major spine surgery. Neurosurg. Rev..

[22] Zale C.L., McIntosh A.L. (2022). Adolescent idiopathic scoliosis for pediatric providers. Pediatr. Ann..

[23] Taenzer A.H., Clark C.J.P.A. (2010). Efficacy of postoperative epidural analgesia in adolescent scoliosis surgery: A meta-analysis. Paediatr. Anaesth..

[24] Slinchenkova K., Lee K., Choudhury S., Sundarapandiyan D., Gritsenko K. (2023). A review of the paravertebral block: Benefits and complications. Curr. Pain. Headache Rep..

[25] Cowie B., McGlade D., Ivanusic J., Barrington M.J. (2010). Ultrasound-guided thoracic paravertebral blockade: A cadaveric study. Anesth. Analg..

[26] Marhofer D., Marhofer P., Kettner S.C., Fleischmann E., Prayer D., Schernthaner M., Lackner E., Willschke H., Schwetz P., Zeitlinger M. (2013). Magnetic resonance imaging analysis of the spread of local anesthetic solution after ultrasound-guided lateral thoracic paravertebral blockade: A volunteer study. Anesthesiology.

[27] Yeying G., Liyong Y., Yuebo C., Yu Z., Guangao Y., Weihu M., Liujun Z. (2017). Thoracic paravertebral block versus intravenous patient-controlled analgesia for pain treatment in patients with multiple rib fractures. J. Int. Med. Res..

[28] Singh N.P., Makkar J.K., Kuberan A., Guffey R., Uppal V. (2022). Efficacy of regional anesthesia techniques for postoperative analgesia in patients undergoing major oncologic breast surgeries: A systematic review and network meta-analysis of randomized controlled trials. Can. J. Anaesth..

[29] Naganuma M., Tokita T., Sato Y., Kasai T., Kudo Y., Suzuki N., Masuda S., Nagaya K. (2022). Efficacy of preoperative bilateral thoracic paravertebral block in cardiac surgery requiring full heparinization: A propensity-matched study. J. Cardiothorac. Vasc. Anesth..

[30] Feray S., Lubach J., Joshi G.P., Bonnet F., Van de Velde M. (2022). Prospect guidelines for video-assisted thoracoscopic surgery: A systematic review and procedure-specific postoperative pain management recommendations. Anaesthesia.

[31] Ardon A.E., Lee J., Franco C.D., Riutort K.T., Greengrass R.A. (2020). Paravertebral block: Anatomy and relevant safety issues. Korean J. Anesthesiol..

[32] Page E.A., Taylor K.L. (2017). Paravertebral block in paediatric abdominal surgery-a systematic review and meta-analysis of randomized trials. Br. J. Anaesth..

[33] Fan Q., Liu H., Li Y., Dai H., Wang Y. (2023). Comparison of ultrasound-guided erector spinae plane block and thoracic paravertebral block for postoperative analgesia after laparoscopic nephrectomy: A randomized controlled non-inferiority clinical trial. Minerva Anestesiol..

[34] Feng J., Wang H., Peng L., Xu H., Song X. (2023). Effects of thoracic paravertebral block on postoperative analgesia in infants and small children undergoing ultra-fast track cardiac anesthesia: A randomized controlled trial. J. Cardiothorac. Vasc. Anesth..

[35] Vecchione T., Zurakowski D., Boretsky K. (2016). Thoracic paravertebral nerve blocks in pediatric patients: Safety and clinical experience. Anesth. Analg..

[36] Alansary A.M., Aziz M.M., Elbeialy M.A.K. (2023). Dexamethasone plus bupivacaine versus bupivacaine in bilateral transincisional paravertebral block in lumbar spine surgeries: A randomized controlled trial. Clin. J. Pain..

[37] Melvin J.P., Schrot R.J., Chu G.M., Chin K.J. (2018). Low thoracic erector spinae plane block for perioperative analgesia in lumbosacral spine surgery: A case series. Can. J. Anaesth..

[38] Zhang J.J., Zhang T.J., Qu Z.Y., Qiu Y., Hua Z. (2021). Erector spinae plane block at lower thoracic level for analgesia in lumbar spine surgery: A randomized controlled trial. World J. Clin. Cases.

[39] Liu H., Zhu J., Wen J., Fu Q. (2023). Ultrasound-guided erector spinae plane block for postoperative short-term outcomes in lumbar spine surgery: A meta-analysis and systematic review. Medicine.

[40] Chenesseau J., Fourdrain A., Pastene B., Charvet A., Rivory A., Baumstarck K., Bouabdallah I., Trousse D., Boulate D., Brioude G. (2023). Effectiveness of surgeon-performed paravertebral block analgesia for minimally invasive thoracic surgery: A randomized clinical trial. JAMA Surg..

[41] Moorthy A., Eochagáin A.N., Dempsey E., Wall V., Marsh H., Murphy T., Fitzmaurice G.J., Naughton R.A., Buggy D.J. (2023). Postoperative recovery with continuous erector spinae plane block or video-assisted paravertebral block after minimally invasive thoracic surgery: A prospective, randomised controlled trial. Br. J. Anaesth..

[42] Bosenberg A., Thomas J., Lopez T., Lybeck A., Huizar K., Larsson L.E. (2002). The efficacy of caudal ropivacaine 1, 2 and 3 mg × L^−1^ for postoperative analgesia in children. Paediatr. Anaesth..

