# Supplementary material for: Effect of Surgeon-Performed Thoracic Paravertebral Block on Postoperative Pain in Adolescent Idiopathic Scoliosis Surgery: A Prospective Randomized Controlled Trial
Source: J Pers Med. 2024 Jun 20;14(6):659. doi: 10.3390/jpm14060659 (PMC11204895; doi:10.3390/jpm14060659)
Supplement: Supplementary file 1 [file jpm-14-00659-s001.zip › jpm-3028456-supplementary.pdf]

**Supplemental Table S1. Demographic and operative variables**

| | Control group (N=16) | PVB group (N=15) | *P* value | Effect size (95% CI) | Power | Statistics | df |
|---|---|---|---|---|---|---|---|
| Age (years) | 14.1 ± 2.6 | 13.7 ± 1.9 | 0.694 | 0.143 (-0.670, 0.850) | 0.067 | 0.398 | 29 |
| Female/Male | 11/5 | 11/4 | >0.999 | 0.050 (0.000, 0.380) | 0.040 | NA | NA |
| Height (cm) | 156.6 ± 11.4 | 160.9 ± 13.0 | 0.332 | -0.354 (-1.210, 0.400) | 0.159 | -0.986 | 29 |
| Weight (kg) | 48.4 ± 8.7 | 52.5 ± 14.8 | 0.364 | -0.336 (-1.050, 0.370) | 0.147 | -0.926 | 22.3 |
| Body mass index (kg/m2) | 19.7 ± 2.6 | 20.1 ± 4.1 | 0.764 | -0.109 (-0.860, 0.770) | 0.060 | -0.303 | 29 |
| ASA class (I/II/III) | 8/6/2 | 10/3/2 | 0.686 | 0.196 (0.000, 0.494) | NA | NA | NA |
| Preoperative Cobb angle | 56.5 [51.5-71.0] | 59.0 [55.5-61.5] | 0.566 | 0.107 (0.007, 0.460) | 0.050 | 105 | NA |
| Preoperative bending | 30.9 ± 15.5 | 30.9 ± 16.0 | 0.999 | 0.001 (-0.690, 0.770) | 0.050 | 0.001 | 29 |
| Flexibility | 48.8 ± 26.5 | 50.2 ± 23.1 | 0.870 | -0.059 (-0.730, 0.660) | 0.053 | -0.166 | 29 |
| Duration of surgery (min) | 249.0 [196.0-354.0] | 273.0 [246.5-300.5] | 0.406 | 0.153 (0.007, 0.500) | 0.073 | 99 | NA |
| Surgical vertebral level during surgery | 8.0 [7.0-13.0] | 11.0 [9.0-11.0] | 0.425 | 0.147 (0.008, 0.510) | 0.111 | 100 | NA |
| Postoperative Cobb angle | 20.2 ± 11.0 | 20.0 ± 9.7 | 0.947 | 0.024 (-0.660, 0.790) | 0.050 | 0.067 | 29 |
| Correction rate | 66.6 ± 18.6 | 67.6 ± 13.7 | 0.862 | -0.063 (-0.820, 0.580) | 0.053 | -0.175 | 29 |
| Anesthesia duration (min) | 340.0 [300.0-457.5] | 375.0 [340.0-417.5] | 0.313 | 0.185 (0.007, 0.510) | 0.069 | 94 | NA |
| Remifentanil use (mcg) | 2694.6 ± 826.9 | 2856.4 ± 1231.2 | 0.687 | -0.158 (-0.950, 0.820) | 0.071 | -0.408 | 25 |
| Propofol use (mg) | 2113.0 [1908.5-2315.0] | 2110.0 [1910.0-3305.5] | 0.478 | 0.161 (0.008, 0.580) | 0.379 | 49 | NA |
| Norepinephrine use (mcg) | 18.5 [4.4-36.0] | 36.2 [24.7-63.5] | 0.163 | 0.285 (0.020, 0.630) | 0.096 | 51 | NA |
| Amount of fluid infused (mL) | 2342.5 ± 794.2 | 2684.7 ± 832.0 | 0.251 | -0.421 (-1.280, 0.260) | 0.205 | -1.172 | 29 |
| Amount of bleeding (mL) | 456.4 ± 245.4 | 686.7 ± 336.2 | 0.037 | -0.787 (-1.620, -0.120) | 0.562 | -2.189 | 29 |
| Amount of transfusion (mL) | 252.4 ± 254.9 | 394.0 ± 160.4 | 0.077 | -0.660 (-1.700, 0.040) | 0.427 | -1.837 | 29 |

Values are reported as the median (interquartile range), mean ± standard deviation, or number of patients (%). ASA, American Society of Anesthesiologists; CI, confidence interval; df, degrees of freedom; NA, not applicable.

**Supplemental Table S2. Intravenous patient-controlled and postoperative rescue analgesia**

| | Control group (N=16) | PVB group (N=15) | *P* value | Effect size (95% CI) | Power | Statistics | df |
|---|---|---|---|---|---|---|---|
| Fentanyl consumption (µg) as background infusion plus boluses via PCA | | | | | | | |
| 0–1 h | 20.3 ± 9.9 | 16.5 ± 10.2 | 0.313 | 0.375 (-0.300, 1.180) | 0.172 | 1.027 | 28 |
| 1–6 h | 78.2 [57.2–92.5] | 96.7 [64.8–130.3] | 0.338 | 0.177 (0.007, 0.540) | 0.312 | 95 | NA |
| 6–12 h | 72.5 [49.9–107.5] | 67.7 [51.0–121.3] | 0.859 | 0.036 (0.007, 0.420) | 0.102 | 115 | NA |
| 12–24 h | 133.8 [95.5–212.4] | 125.7 [94.1–211.8] | 0.953 | 0.014 (0.007, 0.390) | 0.064 | 122 | NA |
| 24–48 h | 282.2 ± 147.7 | 291.8 ± 81.1 | 0.823 | -0.080 (-0.910, 0.590) | 0.055 | -0.226 | 23.6 |
| Number of boluses given via PCA | | | | | | | |
| 0–1 h | 2 (1–3) | 1 (0–2) | 0.050 | 0.364 (0.040, 0.670) | 0.523 | 148.5 | NA |
| 1–6 h | 6.6 ± 3.4 | 8.3 ± 4.4 | 0.237 | -0.441 (-1.200, 0.280) | 0.220 | -1.207 | 28 |
| 6–12 h | 5.0 [2.5–8.0] | 4.0 [1.5–9.5] | 0.983 | 0.008 (0.004, 0.420) | 0.052 | 113.5 | NA |
| 12–24 h | 8.0 [2.5–20.0] | 5.0 [3.0–13.0] | 0.708 | 0.072 (0.004, 0.440) | 0.082 | 122 | NA |
| 24–48 h | 13.1 ± 11.9 | 13.7 ± 8.3 | 0.886 | -0.06 (-1.080, 0.880) | 0.053 | -0.145 | 22 |
| Number of invalid bolus attempts via PCA | | | | | | | |
| 0–1 h | 3.5 [0.0–6.0] | 1.0 [0.0–3.0] | 0.267 | 0.210 (0.009, 0.570) | 0.101 | 130 | NA |
| 1–6 h | 3.0 [0.5–11.5] | 1.0 [0.0–10.5] | 0.556 | 0.111 (0.008, 0.460) | 0.059 | 127 | NA |
| 6–12 h | 0.0 [0.0–4.0] | 0.0 [0.0–6.0] | 0.982 | 0.008 (0.004, 0.390) | 0.113 | 113.5 | NA |
| 12–24 h | 1.0 [0.0–10.5] | 0.0 [0.0–4.0] | 0.485 | 0.008 (0.004, 0.390) | 0.113 | 113.5 | NA |
| 24–48 h | 1.0 [0.0–3.0] | 0.0 [0.0–3.0] | 0.804 | 0.057 (0.006, 0.460) | 0.170 | 76 | NA |
| Patients receiving rescue analgesics (n) | | | | | | | |
| 0–1 h | 13 (81%) | 10 (67%) | 0.433 | 0.167 (0.000, 0.518) | 0.120 | NA | NA |
| 1–6 h | 7 (44%) | 7 (47%) | >0.999 | 0.029 (0.000, 0.327) | 0.053 | 0.000 | 1 |
| 6–12 h | 2 (13%) | 5 (33) | 0.220 | 0.249 (0.000, 0.601) | 0.290 | NA | NA |
| 12–24 h | 3 (19%) | 6 (40%) | 0.252 | 0.234 (0.000, 0.586) | 0.180 | NA | NA |
| 24–48 h | 6 (38%) | 10 (67%) | 0.206 | 0.292 (0.000, 0.644) | 0.369 | 1.599 | 1 |

Values are presented as median (interquartile range), mean ± standard deviation, or number of patients (%). CI, confidence interval; df, degrees of freedom; NA, not applicable; PCA, intravenous patient-controlled analgesia.

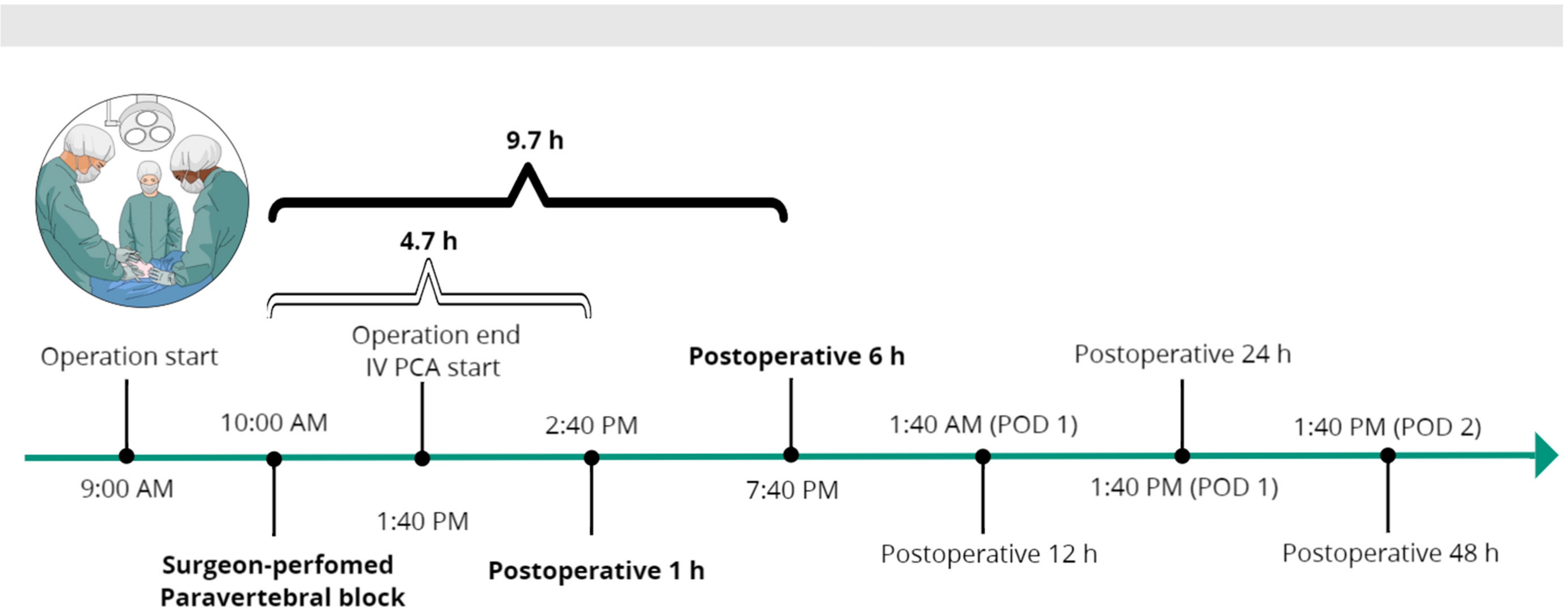


**Figure S1**. Study timeline.

***Effect Size**

For an independent t-test, Cohen's d is used. For the Mann-Whitney U test, Wilcoxon effect size (r) is used. For Fisher's exact test or chi-square test, Cramer's V is calculated.

Cohen's |d| values are considered small for $0.2 \leq d < 0.5$, medium for $0.5 \leq d < 0.8$, and large for $d \geq 0.8$.

Wilcoxon effect size (r) values are considered small for $0.1 \leq r < 0.3$, moderate for $0.3 \leq r < 0.5$, and large for $r \geq 0.5$.

Cramer's V values are considered small for $0.1 \leq V < 0.3$, medium for $0.3 \leq V < 0.5$, and large for $V \geq 0.5$.

(Reference: Cohen J. Statistical Power Analysis for the Behavioral Sciences. Lawrence Erlbaum; 1988.)

***Statistics and Degrees of Freedom**

For an independent t-test, report the t statistic and degrees of freedom (df).

For a chi-square test, report the $\chi^2$ statistic and degrees of freedom (df).

For a Mann-Whitney U test, since it does not use approximation but exact distribution for p-value, report the U-value and denote degrees of freedom (df) as not applicable (NA).

For a Fisher exact test, since it does not use approximation but exact distribution for p-value, denote the statistic as NA and degrees of freedom (df) as NA.
